# Pars Plana Vitrectomy versus Intravitreal Injection of Ranibizumab in the Treatment of Diabetic Macular Edema Associated with Vitreomacular Interface Abnormalities

**DOI:** 10.1155/2021/6699668

**Published:** 2021-01-12

**Authors:** Hassan Elkayal, Ahmed M. Bedda, Hesham El-Goweini, Ahmed A. Souka, Amir Ramadan Gomaa

**Affiliations:** Ophthalmology Department, Faculty of Medicine, Alexandria University, Alexandria, Egypt

## Abstract

**Purpose:**

To compare the efficacy of pars plana vitrectomy (PPV) versus intravitreal injection (IVI) of ranibizumab (RBZ) in the treatment of diabetic macular edema (DME) associated with vitreomacular interface abnormalities (VMIA).

**Methods:**

The records of patients presenting with DME and VMIA throughout 2016 to 2018 were retrospectively analyzed. The patients were divided into 2 groups: group I received IVIs of RBZ and group II underwent PPV with internal limiting membrane peeling. The main outcome measures were the change in the LogMAR corrected distance visual acuity (CDVA) and central subfield thickness (CSFT) on optical coherence tomography over 6 months.

**Results:**

At 6 months, mean CDVA improved by 0.22 ± 0.21 in group I patients (*p* < 0.001), while in group II, it improved only by 0.09 ± 0.22 (*p* < 0.115). Fifty-five percent of group I and 60% of group II patients had stable CDVA (within 2 lines from baseline) at 6 months. Significant improvement in vision (gain of 2 or more lines) was seen in 45% and 30%, respectively. Worsening of vision (loss of 2 or more lines) was seen only in 2 patients in group II, but none in group I. The mean CSFT improved significantly in both groups (by 162 *µ* and 216 *µ*, respectively; *p* < 0.001). The mean CSFT at 6 months was similar in both groups (354 *µ* and 311 *µ*, respectively; *p*=0.172).

**Conclusions:**

Both treatments resulted in anatomical improvement of DME with concurrent VMIA. Visual improvement was more pronounced in the IVI group, although this may have been affected by other confounding factors.

## 1. Introduction

Diabetic macular edema (DME) is the most common cause of moderate visual loss in diabetic patients [1, 2]. The posterior hyaloid (PH) and vitreomacular interface abnormalities (VMIA) play a role in the pathogenesis of DME. It has been demonstrated that there is a high prevalence of an attached or partially attached PH in cases of DME and that the spontaneous release of the PH is associated with resolution of DME [3–5]. VMIA tend to occur in about 6–22% of eyes with DME [6–8]. VMIA include epiretinal membrane (ERM) and vitreomacular traction (VMT) [9, 10]. Vitreomacular adhesion is considered a normal finding in the natural course of posterior vitreous detachment (PVD) and is not graded as part of VMIA [9].

Intravitreal injection (IVI) of anti-vascular endothelial growth factor (anti-VEGF) agents has become the mainstay of treatment for nontractional DME [11]. VMIA have been shown in some studies to be predictive of a reduced therapeutic effect to anti-VEGF injections [12, 13], while other studies found no association [14].

The treatment of primarily tractional DME is essentially surgical. Pars plana vitrectomy (PPV) with removal of all tractional elements including the PH with or without internal limiting membrane (ILM) peeling is the procedure employed, resulting in both anatomical and visual improvement in many cases [15, 16]. The role of PPV in nontractional refractory DME is controversial, with only some studies showing visual and anatomical improvement [17–19].

The aim of this work was to compare the efficacy of PPV versus IVIs of ranibizumab (RBZ) (Lucentis, Novartis) in the treatment of patients with DME associated with VMIA by determining the change in corrected distance visual acuity (CDVA) and central subfield thickness (CSFT) on optical coherence tomography (OCT) over a period of 6 months.

## 2. Subjects and Methods

The study was designed to be a retrospective comparative trial. The records of patients presenting with DME associated with VMIA to the Ophthalmology Department of Alexandria University between January 2016 and December 2018 were reviewed. The study was approved by the Ethics Committee of Alexandria University and adhered to the Tenets of the Declaration of Helsinki.

### 2.1. Inclusion Criteria

(1) A confirmed diagnosis of diabetes mellitus.
(2) Age ≥ 18 years.
(3) CDVA between and including 6/9 and 6/120 (measured by a Snellen chart).
(4) A diagnosis of DME associated with VMIA:
  - DME with CSFT > 305 *µ*m in females or CSFT > 320 *µ*m in males as measured by the Spectralis OCT (Heidelberg Engineering, Heidelberg, Germany)
  - VMIA (VMT, ERM, or both) detected by the Spectralis OCT and defined below.
(5) Patients receiving treatment in the form of IVIs of RBZ (group I) or PPV (group II). The choice of treatment methods was determined by the treating clinician.
(6) Follow-up data available for 6 months during which the patient did not receive other alternative treatments for DME. The use of a postoperative course of steroid eye drops and nonsteroidal anti-inflammatory drops was allowed.

### 2.2. Exclusion Criteria

1. Ocular diseases other than diabetic retinopathy including significant cataract, glaucoma, or uveitis.
2. Active proliferative diabetic retinopathy (PDR) requiring prompt panretinal photocoagulation (PRP) (patients with PRP performed >3 months could still be included).
3. Vitreous hemorrhage.
4. Severe central ischemic maculopathy defined as the foveal avascular zone (FAZ) >1000 *µ*m in diameter and a completely destroyed FAZ capillary outline [20].
5. Advanced diabetic eye disease with tractional retinal elevation involving the macula (including tractional retinal detachment or tractional retinoschisis).
6. Recent previous treatment for DME (including IVIs of anti-VEGF or grid laser in the last 3 months, or IVIs of steroids in the last 6 months).
7. Previous PPV.
8. Previous cataract surgery within the last 3 months.

### 2.3. OCT Imaging

Two independent reviewers graded the OCT images to confirm the presence and subtype of VMIA. Only patients in which both reviewers agreed to the presence of DME with concurrent VMIA were included in the study.

### 2.4. OCT Patterns

(1) Vitreomacular traction (VMT): The international vitreomacular interface study (IVTS) group defined VMT by the following criteria being present on at least 1 B-scan on OCT:
  - Perifoveal PVD.
  - Persistent vitreous attachment in a 6 mm-diameter circle centered around the fovea.
  - Association of the attached vitreous with retinal anatomic changes at the site of vitreous attachment (distortion of foveal surface, intraretinal cysts, elevation of the foveal floor, or a combination of them).
VMT can be focal (≤1500 *µ*m) or broad (>1500 *µ*m) [9].
(2) Epiretinal membrane (ERM): ERM is seen as a hyper-reflective band along the inner retinal surface. It can be partially adherent or globally adherent to the retinal surface [10].
(3) Both VMT and ERM.

### 2.5. Group I

Patients received IVIs of 0.5 mg/0.05 mL RBZ. The injection protocol was determined by the treating clinician.

### 2.6. Group II

Patients underwent standard 23-gauge PPV with induction of PVD and complete removal of the vitreous gel. This was followed by ERM and ILM peeling assisted with the use of brilliant blue G 0.025%.

### 2.7. Outcome Measures

Primary outcome measures were the mean change in CDVA and CSFT at 6 months, and the secondary outcomes were postoperative complications (such as significant cataract, high IOP >30 mmHg not controlled by eye drops, vitreous hemorrhage, retinal detachment, and endophthalmitis).

### 2.8. Statistical Analysis of the Data

CDVA was converted to LogMAR units for statistical purposes. Statistical analysis was performed using IBM SPSS software package, version 20.0. (Armonk, NY: IBM Corp). Qualitative data were described using number and percent. The Kolmogorov–Smirnov test was used to verify the normality of distribution. Quantitative data were described using mean ± standard deviation. Significance of the obtained results was judged at the 5% level.

## 3. Results

A total of 40 patients were included from the records, 20 of which received IVIs of RBZ (group I) and 20 underwent PPV (group II).  Table 1 shows the baseline characteristics of the patients in both groups. Most characteristics were well balanced between the two groups, but some differences were noted:

1. More patients in group II had more advanced stages of diabetic retinopathy as compared with group I (*p*=0.047, chi-squared test). Quiescent PDR was present in only 20% of patients in group I compared with 60% in group II.
2. Group I had 13 treatment naïve patients as compared with only 5 in group II (*p*=0.024). On the other hand, there were 7 patients in group II who had previous macular laser treatment as compared with a single patient in group I (*p*=0.024).
3. There was a larger proportion of phakic patients in group I (60%) as compared with 25% in group II (*p*=0.025).

**Figure 1:**
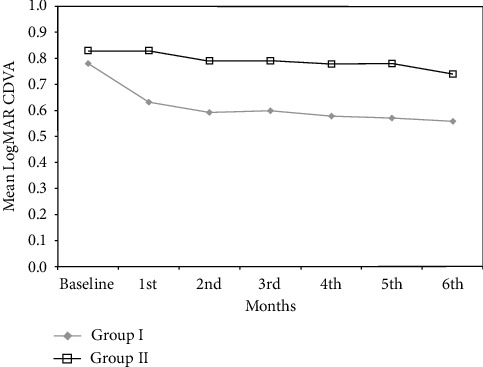
Change in the mean corrected distance visual acuity in the two groups.

At baseline, there was no significant difference between the 2 groups in the mean LogMAR CDVA. From months 1 to 5, the CDVA became significantly better in group I compared with group II (*p* < 0.05, Student's *t*-test) (Table 2, Figure 1). At 6 months, mean CDVA in group I was 0.56 ± 0.29 compared with 0.74 ± 0.29 in group II. Although the difference in the mean CDVA between the 2 groups was 0.18 LogMAR at 6 months, it did not reach statistical significance (*p*=0.061).

Also, when assessing the change in vision as a categorical value, the differences between both groups were not statistically significant (*p*=0.351, chi-squared test) (Table 3). More than half of the patients in each group had stable CDVA (within 2 lines from baseline) at the end of the study. Significant improvement in vision (2 or more lines) was seen in 45% of patients in group I and 30% of those in group II. Worsening of vision (loss of 2 or more lines) was seen only in 2 patients in group II but none in group I.

A difference between the groups was observed when assessing the change in the mean CDVA within each group. CDVA significantly improved during the 6 months of the study by 0.22 ± 0.21 in group I patients (*p* < 0.001, ANOVA test). In group II, on the other hand, mean CDVA did not improve significantly with the mean CDVA improving by 0.09 ± 0.22 (*p* = 0.115).

**Figure 2:**
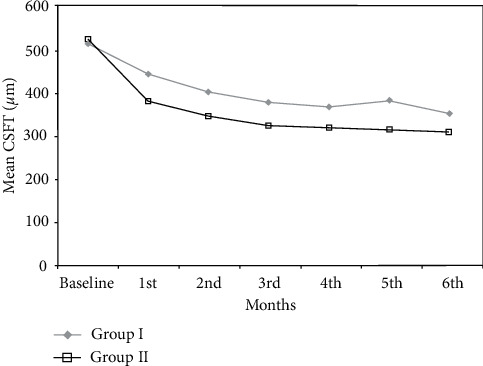
Change in the mean central subfield thickness in the two groups.

Baseline mean CSFT was similar in both groups (516 *µ*m ± 93 in group I versus 527 *µ*m ± 116 in group II, *p*=0.759), and both groups showed a significant reduction of CSFT during the study period (Table 2, Figure 2). In group I, mean CSFT decreased to 354 *µ*m ± 105 at 6 months (*p* < 0.001). In group II, mean CSFT decreased to 311 *µ*m ± 94 at 6 months (*p* < 0.001). When comparing the 2 groups, the final CSFT was not statistically significant (*p*=0.172). A significantly lower CSFT in group II as compared with group I was only present in months 1 and 5 of the study (*p*=0.036 and 0.038, respectively).

**Figure 3:**
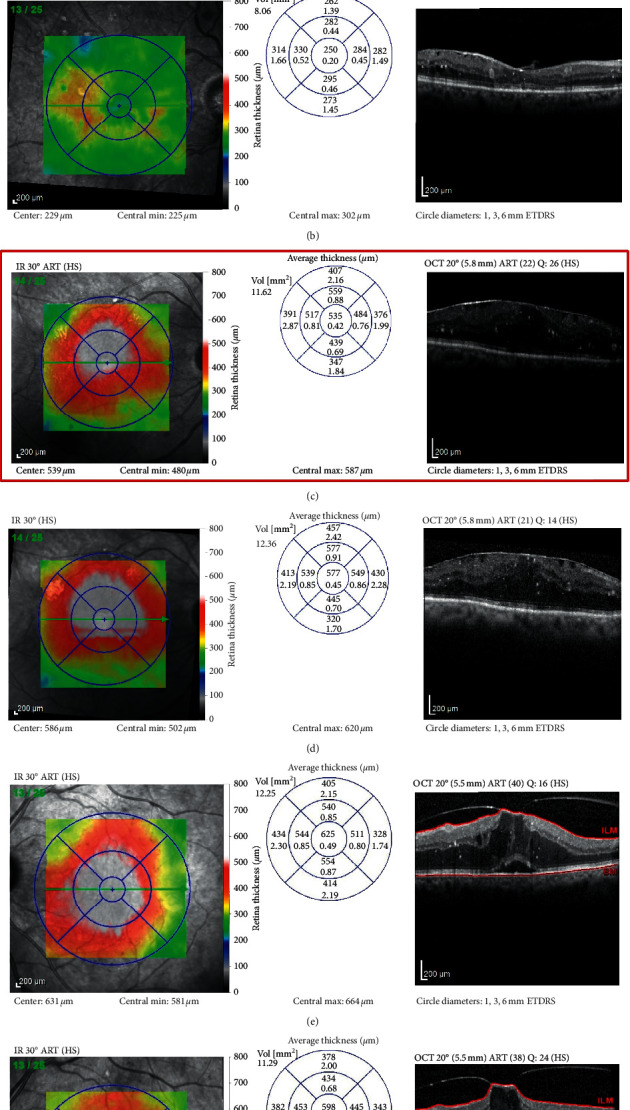
Sample cases from group I. Case 1: (a) baseline OCT scan of DME with concurrent ERM, CDVA 0.3; and (b) OCT scan at the end of the study after 4 IVIs of RBZ with resolution of the central DME, CDVA improved to 0.18. Case 2: (c) baseline OCT scan of DME with concurrent ERM, CDVA 0.6; and (d) OCT scan after 6 IVIs of RBZ with persistent nonresponding DME, CDVA 0.7. Case 3: (e) baseline OCT scan of DME with concurrent focal VMT, CDVA 0.6; and (f) OCT scan after 6 IVIs of RBZ partial improvement in parafoveal edema, progression of VMT, and persistent central DME, CDVA 0.6.

**Figure 4:**
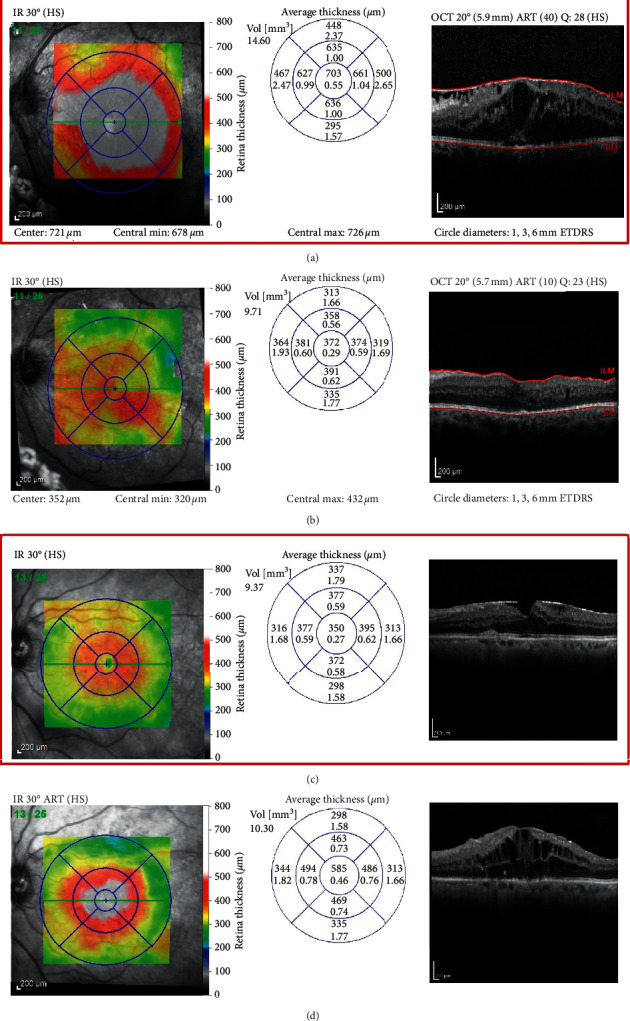
Sample cases from group II. Case 1: (a) baseline OCT scan of DME with concurrent ERM, CDVA 1.0; and (b) OCT scan 6 months after PPV showing marked improvement in central macular thickness, CDVA improved to 0.77. Case 2: (c) baseline OCT scan of DME with concurrent ERM, CDVA 0.82; and (d) OCT scan 6 months after PPV showing worsening of cystoid macular edema, CDVA dropped to 0.92.

Seven out of 20 patients achieved a dry macula at the end of the study in group I as compared with 11/20 in group II. Improvement in macular thickness (reduction of CSFT ≥10% of baseline) was noted in 11/20 patients in group I and in 8/20 in group II. Two patients in group I had persistent DME (CSFT change <10% of baseline) (Figure 3), while 1 patient in group II had worsening of DME (increase in CSFT ≥10% of baseline) (Figure 4). Overall, the differences between both groups was not statistically significant (*p*=0.208).

Eighty five percent of the patients receiving RBZ (17/20 patients) had no change to their VMIA status. One patient with VMT showed progression and worsening of traction with persistent DME and stable VA. Another patient with VMT showed complete PVD with resolution of traction and complete resolution of DME and was one of two patients requiring only 3 IVIs during the 6 months of the study. A third patient with a combined ERM and VMT  showed partial release of VMT after receiving injections.

The mean number of IVIs for group I patients was 5.1 ± 1.1. The minimum number of IVIs was 3 (*n* = 2), and the maximum was 6 (*n* = 11). There were no major complications observed in either group. Only 1 phakic patient out of 5 in group II had progression to a moderately dense nuclear cataract at the 6-month endpoint of the study.

## 4. Discussion

The cause of macular edema in a diabetic patient with concurrent VMIA can be multifactorial. It is likely a mixture of mechanical traction and capillary hyperpermeability secondary to the microvascular alterations associated with the metabolic abnormalities of diabetes. It is sometimes difficult to determine which factor predominates, and there is no consensus on the best treatment approach in these cases. The current study attempted to compare both treatment options, namely IVI of anti-VEGF and PPV.

In both groups, ERM was the most common form of VMIA (62.5% of the whole cohort), while the least common form was having both an ERM and VMT (12.5% of the whole cohort). This pattern is similar to what was reported in other studies [14, 21].

### 4.1. Outcomes of IVIs of Anti-VEGF in DME with Concurrent VMIA

In the current study, group I patients treated with RBZ had a significant improvement in CDVA (a mean improvement of 0.22 LogMAR) and 45% had 2 or more lines of improvement in vision. They also had a significant reduction in CSFT on OCT (a mean reduction of 162 *µ*m) with 35% achieving a dry macula at 6 months.

Previous studies of anti-VEGF in DME with concurrent VMIA had variable results.

A post hoc analysis of the DRCR.net Protocol I results concluded that DME patients with surface wrinkling on fundus photos had less improvement in vision and less reduction of CSFT on OCT [22]. Similarly, a small study by Wu et al. concluded that DME patients with VMIA had a less favourable response, visually and anatomically, 1 month after a single IVI of BVZ [12, 23].

A larger prospective study by Wong et al. included 104 eyes with DME treated by IVIs of RBZ over a year. A clinically significant ERM at baseline was predictive of worse final visual and anatomic outcomes [13]. On the other hand, Mikhail et al. did not find an association between VMIA and visual or anatomic outcomes after injections. They retrospectively reviewed the records of 146 eyes with DME who were treated with IVIs of RBZ and followed for a mean of 9 months. Patients with VMIA at baseline (according to the IVTS criteria) presented with lower visual acuity; nevertheless, the presence of VMIA did not reduce the response to treatment with RBZ [14].

### 4.2. Outcomes of PPV in DME with Concurrent VMIA

In the current study, group II patients undergoing PPV were not observed to have a significant improvement in VA. The mean CDVA improved only by 0.09 LogMAR. Improvement in VA by ≥2 lines was observed in 30% of patients, 10% lost ≥2 lines of VA, and 60% maintained stable vision within 2 lines of baseline vision. On the other hand, a significant reduction in CSFT on OCT was observed in the PPV group with a mean reduction of 216 *µ*m and 55% of patients achieving a dry macula at the end of the study.

The DRCR.net evaluated the outcomes of PPV in 87 eyes having DME with VMT. The presence of VMT was determined by the clinical assessment of the investigator rather than by OCT criteria. ILM peeling was done in 54% of the cases. At 6 months, median VA did not improve significantly (+3 letters compared with baseline). Improvement in VA ≥10 letters was seen in 38%, while 22% had worsening of VA by ≥10 letters and 40% remained stable within 10 letters. There was a significant reduction in CSFT on OCT by 160 *µ*m at 6 months. Around 43% of patients had a CSFT ≤250 *µ*m at 6 months [16].

Another cohort of 20 patients with tractional DME was followed up by Bonnin et al. for a mean of 5.3 years after undergoing PPV with ILM peeling. Patients showed a significant improvement in VA (mean improvement of 0.3 LogMAR) and OCT thickness (mean reduction of 243 *µ*m in CSFT). Improvement in VA by ≥2 lines was seen in 25% of patients, 12.5% lost ≥2 lines of VA, and 62.5% maintained stable VA within 2 lines of baseline VA [17].

A retrospective study by Pessoa et al. reviewed 46 eyes with tractional DME who underwent PPV with ILM peeling. Patients were determined to have VMIA according to the OCT classification of the IVTS. At 12 months, there was a significant improvement in the median VA by 23 letters and a significant reduction of CSFT by 215 *μ*m on OCT. The VA was observed to continue to improve gradually along the 12 months of the study, while the reduction in CSFT occurred mainly in the first 3 months but was minimal thereafter. An improvement in VA by ≥10 letters was achieved in 60% of cases. Worsening by ≥10 letters was seen in 13% of cases, and 27% had stable VA at 12 months [21].

In summary, all studies showed a definite anatomical improvement although this did not always correlate with vision improvement.

### 4.3. Comparison of Outcomes between Anti-VEGF and PPV

The mean CDVA was not different between both groups at baseline. There was an earlier and more prominent improvement in mean CDVA in the IVI group, although at the end of the study the 0.18 LogMAR difference did not reach statistical significance. One explanation is that the PPV group had 2 patients with more than 2 lines loss of VA at 6 months as opposed to none in the IVI group. This may have affected the mean CDVA outcome of the whole PPV group. The cause of visual loss in one of those 2 patients was migrating hard exudates that coalesced into a plaque and was eventually encroaching upon the fovea. The other patient had thinning of the macula on OCT. These atrophic changes in the macula sometimes called the “floor effect” have been previously reported. The proposed theory for that relies on the fact that the ILM is much more adherent to the retina and foot plates of Müller cells in diabetic retinopathy. The peeling of this adherent ILM may induce injury to the Müller cells, resulting in their collapse and subsequently retinal thinning [24].

Also, it might be argued that vision improvement in the PPV group may have been limited by post-PPV cataract progression. The effect of that was probably minimal as only 5 out of 20 patients in group II were phakic at baseline and only 1 of those had progression to a moderately dense nuclear cataract at the end of the study. The presence of a larger proportion of patients with more advanced stages of DR and with a history of previous macular laser treatment in the PPV group at baseline may have limited their potential for visual improvement.

Both groups showed similar reduction in CSFT and had similar rates of patients achieving dry macula or demonstrating improvement in DME. In the IVI group, no patients had worsening of DME, but 2 patients had persistent nonresponsive DME. In the other group, there was one patient with worsening of cystoid macular edema after PPV. Overall, the pattern of improvement of CSFT was that of an earlier reduction in thickness in the PPV group as compared with a more gradual reduction in the IVI group.

The study had some limitations, mainly those inherent to its retrospective design. This includes selection bias and the imbalance in some baseline characteristics between the groups. A prospective randomized controlled trial with a large sample size would allow randomization of any confounding factors and would provide sufficient power to study the treatment response in the different subtypes of VMIA (ERM and VMT) separately. Long-term follow-up data would help determine the longevity of the treatment benefits and the recurrence rate of DME with different types of treatment.

## 5. Conclusions

In conclusion, both treatment options resulted in anatomical improvement of DME. Visual improvement was more pronounced in the IVI group, although this may have been affected by other confounding factors. Both mechanical and biochemical factors contribute to retinal thickening in cases of DME with VMIA. An optimal treatment option is yet to be determined. It might be that no single treatment is best and that a combination between both IVIs and PPV may offer a better option in a certain subgroup of patients with DME and concurrent VMIA as it can address multiple factors in the underlying disease processes.

## Figures and Tables

**Table 1:** Baseline characteristics of the patients in the two groups.

	Group I		Group II		*p* value
	(*n* = 20)		(*n* = 20)		
*Gender*					
Male	8	(40%)	6	(30%)	*p*=0.507
Female	12	(60%)	14	(70%)	

*Age (years)*	67 ± 8		63 ± 11		*p*=0.233

*Diabetes*					
Type 1	0	(0%)	1	(5%)	*p*=1.000
Type 2	20	(100%)	19	(95%)	

*Diabetic retinopathy*					
Moderate NPDR	10	(50%)	5	(25%)	*p*=0.047^*∗*^
Severe NPDR	6	(30%)	3	(15%)	
Quiescent PDR	4	(20%)	12	(60%)	

*HbA1c (%)*	8.1 ± 0.4		8.2 ± 0.3		*p*=0.169

*Previous treatment*					
None	13	(65%)	5	(25%)	*p*=0.024^*∗*^
IVIs of anti-VEGF/steroids	6	(30%)	8	(40%)	
Macular laser	0	(0%)	4	(20%)	
Both	1	(5%)	3	(15%)	

*VMIA*					
ERM	14	(70%)	11	(55%)	*p*=0.645
VMT	4	(20%)	6	(30%)	
ERM and VMT	2	(10%)	3	(15%)	

*Subtype of VMIA*					
*ERM*					*p*=0.680
Partially adherent	8/16	(50%)	9/14	(64%)	
Globally adherent	8/16	(50%)	5/14	(36%)	
*VMT*					
Broad	1/6	(17%)	3/9	(33%)	
Focal	5/6	(83%)	6/9	(67%)	

*Lens*					
Phakic	12	(60%)	5	(25%)	*p*=0.025^*∗*^
Pseudophakic	8	(40%)	15	(75%)	

*CDVA (LogMAR)*	0.78 ± 0.29		0.83 ± 0.28		*p*=0.618
*CSFT (µm)*	516 ± 93		527 ± 116		*p*=0.759

^*∗*^Statistically significant at *p* ≤ 0.05. Group I: ranibizumab; Group II: pars plana vitrectomy; NPDR: nonproliferative diabetic retinopathy; PDR: proliferative diabetic retinopathy; HbA1c: hemoglobin A1c; IVIs: intravitreal injections; anti-VEGF: anti-vascular endothelial growth factor; VMIA: vitreomacular interface abnormalities; ERM: epiretinal membrane; VMT: vitreomacular traction; CDVA: corrected distance visual acuity; CSFT: central subfield thickness.

**Table 2:** Changes in visual acuity and OCT measurements during the study period.

Months	CDVA (LogMAR)		^*t*^ *p* value	CSFT(*µ*m)		^*t*^ *p* value
	Group I (*n* = 20)	Group II (*n* = 20)		Group I (*n* = 20)	Group II (*n* = 20)	
Baseline	0.78 ± 0.29	0.83 ± 0.28	0.618	516 ± 93	527 ± 116	0.759
1^st^	0.63 ± 0.27	0.83 ± 0.27	0.027^*∗*^	446 ± 107	382 ± 77	0.036^*∗*^
2^nd^	0.59 ± 0.26	0.79 ± 0.24	0.017^*∗*^	404 ± 106	348 ± 83	0.068
3^rd^	0.60 ± 0.27	0.79 ± 0.26	0.032^*∗*^	381 ± 112	325 ± 88	0.090
4^th^	0.58 ± 0.28	0.78 ± 0.25	0.024^*∗*^	370 ± 105	321 ± 90	0.129
5^th^	0.57 ± 0.30	0.78 ± 0.27	0.030^*∗*^	384 ± 106	317 ± 93	0.038^*∗*^
6^th^	0.56 ± 0.29	0.74 ± 0.29	0.061	355 ± 105	311 ± 94	0.172
^*F*^ *p* value	<0.001^*∗*^	0.115		<0.001^*∗*^	<0.001^*∗*^	

Group I: ranibizumab; Group II: pars plana vitrectomy; t: Student's *t*-test; F: F test (ANOVA); CDVA: corrected distance visual acuity; CSFT: central subfield thickness. ^*∗*^Statistically significant at *p* ≤ 0.05.

**Table 3:** Categories of CDVA change.

CDVA	Group I (*n* = 20)		Group II (*n* = 20)		^*MC*^ *p*
	No.	%	No.	%	
Improved ≥2 lines	9	(45)	6	(30)	0.351
Stable within 2 lines	11	(55)	12	(60)	
Lost ≥2 lines	0	(0)	2	(10)	

CDVA: corrected distance visual acuity; Group I: ranibizumab; Group II: pars plana vitrectomy; MC: Monte-Carlo correction of the chi-square test; *p* : *p* value for comparing between the two studied groups.

## Data Availability

The data used to support the findings of this study are available from the corresponding author upon request.

## Additional Points

Intravitreal ranibizumab and pars plana vitrectomy are suitable treatment options for diabetic macular edema with concurrent vitreomacular interface abnormalities. Both provide similar anatomical results, while intravitreal injections seem to offer better visual outcomes on the short term.
